# A pilot cohort study to assess the feasibility of HIV prevention science research among men who have sex with men in Dakar, Senegal

**DOI:** 10.7448/IAS.16.4.18753

**Published:** 2013-12-02

**Authors:** Fatou Maria Dramé, Emily E Crawford, Daouda Diouf, Chris Beyrer, Stefan D Baral

**Affiliations:** 1 Université Gaston Berger/Enda Santé, Dakar, Senegal; 2 Department of Epidemiology, Center for Public Health and Human Rights, John Hopkins Bloomberg School of Public Health, Baltimore, MD, United States; 3 Enda Santé, Dakar, Senegal

**Keywords:** HIV, socio-economic status, men who have sex with men, Africa, prevention

## Abstract

**Introduction:**

Men who have sex with men (MSM) are disproportionately burdened by HIV in Senegal, across sub-Saharan Africa and throughout the world. This is driven in part by stigma, and limits health achievements and social capital among these populations. To date, there is a limited understanding of the feasibility of prospective HIV prevention studies among MSM in Senegal, including HIV incidence and cohort retention rates.

**Methods:**

One hundred and nineteen men who reported having anal sex with another man in the past 12 months were randomly selected from a sampling frame of 450 unique members of community groups serving MSM in Dakar. These men were enrolled in a 15-month pilot cohort study implemented by a community-based partner. The study included a structured survey instrument and biological testing for HIV, syphilis and hepatitis B virus at two time points.

**Results:**

Baseline HIV prevalence was 36.0% (43/114), with cumulative HIV prevalence at study end being 47.2% (51/108). The annualized incidence rate was 16% (8/40 at risk for seroconversion over 15 months of follow-up, 95% confidence interval 4.6–27.4%). Thirty-seven men were lost to follow up, including at least four deaths. Men who were able to confide in someone about health, emotional distress and sex were less likely to be HIV positive (OR 0.36, *p <* 0.05, 95% CI 0.13, 0.97).

**Conclusions:**

High HIV prevalence and incidence, as well as mortality in this young population of Senegalese MSM indicate a public health emergency. Moreover, given the high burden of HIV and rate of incident HIV infections, this population appears to be appropriate for the evaluation of novel HIV prevention, treatment and care approaches. Using a study implemented by community-based organizations, there appears to be feasibility in implementing interventions addressing the multiple levels of HIV risk among MSM in this setting. However, low retention across arms of this pilot intervention, and in the cohort, will need to be addressed for larger-scale efficacy trials to be feasible.

## Introduction

The HIV epidemic in Senegal has followed a pattern distinct from the epidemics observed in Southern and Eastern African countries such as Kenya and Malawi, with a far more concentrated epidemic among key populations such as men who have sex with men (MSM) and female sex workers [1]. The Senegalese government launched an early and comprehensive effort to prevent HIV infection in the general population [2]. This campaign was deemed a success by many and is, in part, likely responsible for the limited HIV epidemic in the country, which reports an HIV prevalence of 0.8% among reproductive age women and 0.5% among men ages 15–49 [3,4]. More recently, there has been increased study of social factors such as unregulated sex work, stigma and discrimination targeting those at high risk of HIV acquisition and transmission, as well as HIV transmission related to same-sex practices among men [5–11].

MSM have multiple, intersecting drivers of risk and have had a consistently higher risk of HIV acquisition and transmission since the first cases of HIV were discovered [1,12]. This disproportionate burden of HIV in MSM has also been observed in Senegal. Studies dating back nearly ten years have highlighted this disproportionate burden with HIV prevalence among MSM reported to be 22.4% in 2004 and 21.8% in 2007 [11]. Based on these and other data, Dramé reported that HIV prevalence among MSM is approximately 50 times higher than the prevalence observed among reproductive age adults in Senegal according to the most recent demographic and health survey [13]. Finally, the attributable fraction of HIV infections among MSM in Senegal is high; Van Griensven *et al*. estimated that nearly a fifth of prevalent HIV infections among men in Senegal are among MSM. Collectively, these data reinforce the need to address the HIV prevention, treatment and care needs of MSM in Senegal [14].

The definition for social capital is “institutions, relationships, attitudes and values that govern interactions among people and contribute to economic and social development” [15]. The importance of social capital has been increasingly recognized as a major social determinant of health because of its association with health outcomes including chronic disease-related morbidity and mortality and, more recently, sexually transmitted infections [16–26]. Specifically, limited social capital has been associated with higher rates of exchange, survival and commercial sex, and associated with a higher burden of HIV among MSM in Africa [27–30]. HIV infection has also been associated with low social capital; this may be particularly relevant for stigmatized groups such as MSM [31–33]. Development of social capital among MSM has been shown to be limited by enacted stigma [34–36]. And discrimination and stigma affecting MSM are well-documented, not only in Senegal, but throughout sub-Saharan Africa and more broadly around the world [6,7]. Niang *et al*. describe the effect that stigma and discrimination can have on health care-seeking behaviours among MSM in Senegal [6]. When men perceive or experience stigma and discrimination in a health care setting, they are less likely to access health services for STI, resulting in higher rates of untreated STI within sexual networks, thereby mediating HIV transmission [37,38].

MSM face additional challenges in countries where sex between men is criminalized [3]. In Senegal, in 2008, several health promoters working in HIV prevention were arrested under suspicion of being homosexual. These arrests, and the fear of further arrests, had wide-ranging effects on HIV in the community of MSM in Senegal [39]. In response, many non-governmental organizations who had been working in the area of HIV prevention among MSM went into hiding for their own safety. Those who continued distributing prevention materials such as condoms and water-based lubricants saw a marked decrease in the number of men accessing their services. The decreased numbers of men accessing services aimed at the community of MSM also resulted in a lesser availability of social support among MSM. Furthermore, and importantly, health care providers began to fear providing services to MSM following the arrests. This had grave implications for many HIV-positive MSM, who were no longer able to access treatment, either because their provider would no longer see them, or because they feared arrest if they left their home. Some have said that these arrests set HIV prevention efforts back ten years [39].

Stigma and discrimination affect HIV risk and social capital not only by affecting how MSM access prevention and treatment, but also by curbing the presence of research and prevention projects targeting this group in Senegal. A small number of research projects in West Africa has resulted in a limited understanding of what interventions work in communities of MSM in this region [37]. Interventions are difficult to implement, particularly given the constrained legal environment [39]. Community-based organizations of MSM are those with the closest ties to the community and the greatest ability to facilitate interventions [40]. However, these organizations are often not legally registered [13]. Despite these significant challenges, HIV prevention interventions have been effectively implemented for MSM in Senegal [11].

For a population where so much information is left unknown, a cohort study can provide relevant data including prospectively measured HIV incidence [41]. This research project had two primary foci. The first was to assess the feasibility of implementing and retaining participants in a community-driven HIV prevention study in Senegal. The second focus was to describe the study participants in terms of HIV and STI prevalence and incidence, risk behaviours and indicators of social capital at baseline.

## Methods

A prospective cohort study of MSM was conducted from June 2011 to October 2012 in Dakar, Senegal, by members of a community-based organization. Researchers worked with MSM Community Organizations to develop a sampling frame composed of 450 unique individual members of all known MSM organizations in Dakar. Men eligible for the study were at least 18 years of age, members of one of the known MSM organizations in Dakar, had lived in Dakar for at least six months and reported having anal sex with another man in the past 12 months.

Ultimately, 119 men were enrolled in the feasibility cohort study. At baseline, all participants completed an informed consent process, a structured survey instrument and a medical examination conducted by an infectious disease physician. The medical examination included a physical exam and syndromic diagnosis and treatment of STI, or a referral for treatment and follow-up if necessary or preferred by the participants. Participants also provided 10 ml of plasma for testing for HIV, hepatitis B and syphilis, according to the Senegalese national testing algorithm [42]. A subset of the participants also received an exploratory intervention. Because of the small sample size and high loss to follow up, the outcomes of this intervention are not statistically relevant and will not be discussed in this paper.

### Follow up

Thirty-seven participants were lost to follow up between T1 (baseline) and T2 (15 months); 14 of these were HIV positive. At the end of the planned implementation period, the remaining participants (*n=*82) again underwent a process of informed consent and completed the same structured survey instrument. At this time period, T2, 60 participants presented for a second session of biological testing for HIV, hepatitis B and syphilis. Whereas at T1, a partner organization was able to perform biological testing on-site immediately following participant surveys, this coordination was not possible at T2. Participants were required to make an additional visit to a clinic for collection of biological samples; 22 participants were unable or unwilling to conduct this additional visit because of inability to pay transportation costs or other competing issues. Retention support was provided by Enda Santé staff through regular visits or phone calls throughout the follow up period, depending on the wishes of the individual.

### Ethics

All human subjects’ research conducted in accordance with this study has been reviewed and approved by the Senegalese National Ethics Committee for Health Research.

### Analytic approaches

The collected data were linked using anonymous codes. Survey data were entered into SPSS, and monitoring data were collected utilizing Microsoft Excel. All data collected were cleaned and merged into a single database. Inconsistencies found during the data cleaning were reconciled to the original questionnaires or laboratory forms.

These data were analyzed using STATA Version 12 (College Station, Texas). Preliminary analysis was conducted using chi square analysis to determine potential associations of social capital at baseline. HIV incidence was calculated by dividing the number of people who seroconverted between T1 and T2 by the number of participants at risk of HIV acquisition (tested negative at T1 and returned for testing at T2), and multiplying this number by person-time. Because of the small sample size and high rate of loss to follow-up, multivariate regression models were not used.

## Results

The cohort consisted of 119 male participants who reported having anal sex with another man in the past 12 months, with ages ranging from 18 to 42 years. The mean age for all participants was 28 years, with half of the participants between the ages of 23 and 32 years. Those who were found to be HIV-infected were older than those who tested HIV negative (*p=*0.05), with an average age of 28.8 (interquartile range: 25, 32), compared to HIV-uninfected MSM who had an average age of 26.5 (interquartile range: 22, 29). All had had some contact with community groups of MSM in Dakar, Senegal. One-third of the participants had a primary school education or less (*n=*43, 36.4%), one-third had attended secondary school (*n=*39, 33.1%), 15.3% (*n=*18) had attended university and an equal percentage (*n=*18, 15.3%) had attended Islamic or Arab schools. A large majority of participants were single (*n=*104, 88.1%), and 77.3% reported living with their family (*n=*92). Table 1 summarizes the demographic, behavioural, social and financial characteristics of the cohort.

**Table 1:** Baseline demographics and other cohort characteristics

	T1	
	
	*n*	%
*N*	119	–
Demographic variables		
Ethnicity		
Wolof	61	48.7
Other	58	48.7
Education		
Primary or less	43	36.4
Secondary	39	33.1
University	18	15.3
Islamic/Arab School	18	15.3
Marital status		
Single	104	88.1
Married (one wife)	9	7.6
Divorced/ separated	5	4.2
Lives with family	92	77.3
Risk variables		
Always uses condom	102	87.1
Always uses condom and water-based lubricant	75	65.2
Has concurrent partnerships with women	91	76.5
Has ever paid for sex	33	27.7
Has had sex for money	60	50.0
Health variables		
Previously tested HIV-positive	20	17.1
Received STI diagnosis at T1	59	49.2
Ever consulted for an STI	54	46.2
Ever tested for HIV	103	88.0
Ever tested for HIV and received results	91	76.5
Social variables		
Relationship with family		
Excellent	51	42.9
Good	41	34.5
OK	20	16.8
Bad	4	3.4
Very bad	3	2.5
Involved in family decisions	92	78.0
Age of first sex with another man		
12 or younger	35	29.4
13–16	26	21.9
17–19	33	27.7
20 or older	25	21.0
Has a confidant	70	60.8
Financial variables		
Can meet all expenses	28	27.2
Has previously received financial support from HIV project	18	15.1
HIV project		
Has professional qualification	57	71.3
Currently has a job	55	68.8

### Retention results

Thirty-seven of 119 participants were lost to follow-up (31.1%), meaning they were unable or unwilling to participate in the study at T2. Fourteen of those lost to follow up were known to be HIV positive. HIV-positive participants were not lost at a significantly different rate than HIV-negative participants (*p=*0.43). No statistically significant differences were found between those lost to follow-up and those retained to Time 2 comparing any of the variables listed in Table 1.

Reasons for loss to follow up include participant death, participants being unreachable via contact information and social networks, or participants having moved outside of Dakar. Of the participants lost to follow up, four are known to have died (4/119, 0.03). Cause of death was not recorded in this study.

### Social capital results

Analysis of social capital was completed using baseline data. Participants reported having contact with a median of two family members. On average, study participants reported being able to meet about half of their living expenses. Seventy participants (60.8%) reported having a confidant, someone to confide in about matters concerning health, emotional distress and sex.

Men who reported having a confidant were less likely to be HIV positive (OR 0.36, *p <* 0.05, 95% confidence interval [CI] 0.13, 0.97). These men were also less likely to report alcohol use (OR 0.22, *p <* 0.01, 95% CI 0.078, 0.64). Men who reported having a confidant and men who are able to meet their expenses were more likely to report using a condom and water-based lubricant at each anal sex act (for confidant: OR 2.50, *p <* 0.05, 95% CI 1.13, 5.51; for expenses: OR 5.11, *p <* 0.05, 95% CI 1.4–19.2). Further results are reported in Table 2.

**Table 2:** Odds of reporting consistent use of condom and water-based lubricant by social capital indicator

	Condom + lubricant use		
	
Social capital indicator	OR	95% CI	*p*
Three or more sex partners each month	0.44	(0.20, 0.98)	0.04
Believes in MSM collective efficacy	0.42	(0.19, 0.91)	0.03
Current job satisfies needs	7.40	(2.69, 20.37)	0.00
Can meet all expenses (compared with those who can meet no expenses)	5.11	(1.36, 19.16)	0.02
Above median ability to meet expenses	2.83	(1.28, 6.24)	0.01
Ever consulted medical care for an STI	0.32	(0.14, 0.75)	0.01

### Biological results

At the baseline medical examination, 49.2% (*n=*59) of participants were diagnosed with an STI. In the biological testing, three cases of syphilis were diagnosed at baseline (prevalence=2.6%), and two cases were diagnosed at follow-up (prevalence=3.3%). Forty-one participants tested HIV-positive at baseline (36.0%). All participants returning for biological testing at T2 were tested for HIV, regardless of prior test results. Sixty-one participants were tested for at T2, 40 of whom had tested negative at baseline. Eight new infections were observed at T2 (15 months follow up), equating to an annualized incidence of 16 cases per 100 person-years (95% CI 4.6–27.4%) (Table 3).

**Table 3:** Observed prevalence and incidence of HIV and STI

	HIV			Syphilis			Hepatitis B		
			
Time	*N*	Frequency	%	*N*	Frequency	%	*N*	Frequency	%
Prevalence									
T1	114	41	36.0	115	3	2.6	115	16	13.9
T2	61	28	45.9	61	2	3.3	61	11	18.0
Cumulative	108	51	47.2	115	5	4.4	115	18	15.7
Incidence (per person-year)									
–	40	8	0.16	61	2	0.03			

## Discussion

This study attempted to use a community based approach to accrue and retain MSM in Senegal for 15 months while implementing a pilot intervention. Although this study was focused on assessing the feasibility of HIV prevention studies, these data also highlight HIV among MSM as an ongoing public health emergency in Senegal. The high incidence of HIV suggests that this is an ideal population in which to assess novel approaches to prevent HIV acquisition. Moreover, the high prevalence of HIV indicates that this is also an ideal population in which to assess the effectiveness of approaches that address the needs of people living with HIV. These approaches would aim to reduce viral load as a means of improving the health of PLHIV, as well as decreasing the risk of onward HIV transmission.

Loss to follow up in this study was significant, which poses a challenge to the success of future HIV prevention research among MSM in Senegal. Reasons for the loss to follow up were likely multifactorial, including the fact that limited resources were appropriated for enhanced retention approaches in this study. In addition, there was a surprisingly high mortality among this group of men that, with a mean age of 28, was relatively young. Although cause of death was not recorded, anecdotal discussions with community members suggested that these deaths were HIV-related. This pilot cohort study leveraged community groups to implement the study rather than academic teams with significant experience in managing cohorts. Thus, the study demonstrates that cohorts are possible using this approach, but that participant retention strategies should be more thoroughly incorporated into the research protocol. Further research, including qualitative research, is needed to better understand characteristics associated with being retained in the study, and there is a need to explore appropriate retention strategies, for example, using linked peer navigators or SMS-based appointment reminder systems.

Traditional HIV prevention interventions, including condom promotion and HIV testing are necessary. But data on the high force of HIV acquisition and transmission among MSM, as well as the high incidence presented here, suggest that these interventions alone are not enough [43]. Addressing the needs of people at high risk for HIV acquisition could be achieved by assessing the feasibility of antiviral-driven measures such as topical or oral chemoprophylaxis. There are currently Phase II rectal microbicide studies for MSM which include a site in South Africa, and these may eventually represent an important strategy [44]. Separately, oral pre-exposure prophylaxis has been shown to be effective among MSM and may represent a relevant strategy for particularly high-risk men with limited condom usage despite exposure to condom promotion programmes [45]. The proportion of participants in this cohort who had previously been tested for HIV was high, 88%, though many had not received their results. This suggests the need to optimize the continuum of HIV care in this population; this should include ensuring that people are first aware of their HIV status, then assessed for treatment eligibility, actively linked to treatment services and provided with adherence support to achieve viral suppression [46].

Given the high prevalence and incidence of HIV, these data suggest the need to evaluate active linkage to care interventions for MSM in Senegal [47]. A recent systematic review of linkage and utilization of HIV medical care among PLHIV in the United States reported several approaches for linkage to care may be efficacious, including counseling, education and health system navigators [48]. This study was comprised of a highly selected and relatively small sample of MSM already linked into community based organizations in Dakar. However, these men are subject to multiple levels of stigma and discrimination, including exclusion from social activities, isolation from broader social networks and a society that has criminalized their behaviour. Thus, effective HIV intervention packages should address the individual biological and behavioural facilitators of HIV acquisition and transmission, but also address the broader structural determinants of HIV affecting these men.

The baseline data suggests a relationship between social capital and HIV risk including sexual practices and, potentially, prevalent HIV infections. Men who had less financial need were significantly more likely to report use of condoms and water-based lubricant. These results are consistent with earlier data from Senegal noting the importance of financial stability integration of social services as part of health services in the country [49]. These data link social capital to HIV-related risks and suggest that addressing sexual risk practices without addressing the social contexts in which they are taking place may have limited benefit [43]. Documentation and anecdotal reports from the past two decades have suggested that the implementation of interventions that address social capital among MSM can potentially effectively decrease marginalization, stigma and the risk for HIV infection [16,17,50–53]. Although the relationship between social capital and HIV risk is complex, increasing trust and community involvement among this vulnerable population may lead to positive changes in social norms and self-efficacy, and can ultimately lead to lower HIV acquisition and transmission risks [16,17,22,23,26,54,55].

The generalizability of this study to the general population of MSM in Senegal is limited for several reasons. Because of the difficulty of contacting MSM, recruitment was conducted using existing community networks allowing for a representative sample of MSM who are members of community organizations in Dakar. However, this approach potentially excluded those who are the most isolated or those who feel the least desire to become involved in the community of MSM. Thus, using a sampling frame derived from members of community based organizations serving MSM potentially selects for a population with higher social capital than average MSM in Senegal. As earlier mentioned, retention in the study was limited, which did not allow for a statistically powered assessment of the benefit of the intervention. Future studies will need to put a heavy focus on participant retention to facilitate evaluation of the tested packages of interventions.

## Conclusions

Moving forward, cohorts of MSM will be needed to characterize the effectiveness of combination HIV prevention approaches in the West African context. The experience of conducting this feasibility cohort study with a pilot financial intervention illustrates the potential feasibility of such studies among MSM in a region where they are known to be at among the highest risk for the acquisition and transmission of HIV.
